# Adaptive learning embedding features to improve the predictive performance of SARS-CoV-2 phosphorylation sites

**DOI:** 10.1093/bioinformatics/btad627

**Published:** 2023-10-17

**Authors:** Shihu Jiao, Xiucai Ye, Chunyan Ao, Tetsuya Sakurai, Quan Zou, Lei Xu

**Affiliations:** Department of Computer Science, University of Tsukuba, Tsukuba 3058577, Japan; Department of Computer Science, University of Tsukuba, Tsukuba 3058577, Japan; Institute of Fundamental and Frontier Sciences, University of Electronic Science and Technology of China, Chengdu, China; Department of Computer Science, University of Tsukuba, Tsukuba 3058577, Japan; Institute of Fundamental and Frontier Sciences, University of Electronic Science and Technology of China, Chengdu, China; Yangtze Delta Region Institute (Quzhou), University of Electronic Science and Technology of China, Quzhou, China; School of Electronic and Communication Engineering, Shenzhen Polytechnic, No. 4089 Shahexi Road, Shenzhen 518000, China

## Abstract

**Motivation:**

The rapid and extensive transmission of the severe acute respiratory syndrome coronavirus 2 (SARS-CoV-2) has led to an unprecedented global health emergency, affecting millions of people and causing an immense socioeconomic impact. The identification of SARS-CoV-2 phosphorylation sites plays an important role in unraveling the complex molecular mechanisms behind infection and the resulting alterations in host cell pathways. However, currently available prediction tools for identifying these sites lack accuracy and efficiency.

**Results:**

In this study, we presented a comprehensive biological function analysis of SARS-CoV-2 infection in a clonal human lung epithelial A549 cell, revealing dramatic changes in protein phosphorylation pathways in host cells. Moreover, a novel deep learning predictor called PSPred-ALE is specifically designed to identify phosphorylation sites in human host cells that are infected with SARS-CoV-2. The key idea of PSPred-ALE lies in the use of a self-adaptive learning embedding algorithm, which enables the automatic extraction of context sequential features from protein sequences. In addition, the tool uses multihead attention module that enables the capturing of global information, further improving the accuracy of predictions. Comparative analysis of features demonstrated that the self-adaptive learning embedding features are superior to hand-crafted statistical features in capturing discriminative sequence information. Benchmarking comparison shows that PSPred-ALE outperforms the state-of-the-art prediction tools and achieves robust performance. Therefore, the proposed model can effectively identify phosphorylation sites assistant the biomedical scientists in understanding the mechanism of phosphorylation in SARS-CoV-2 infection.

**Availability and implementation:**

PSPred-ALE is available at https://github.com/jiaoshihu/PSPred-ALE and Zenodo (https://doi.org/10.5281/zenodo.8330277).

## 1 Introduction

**Figure 1.**
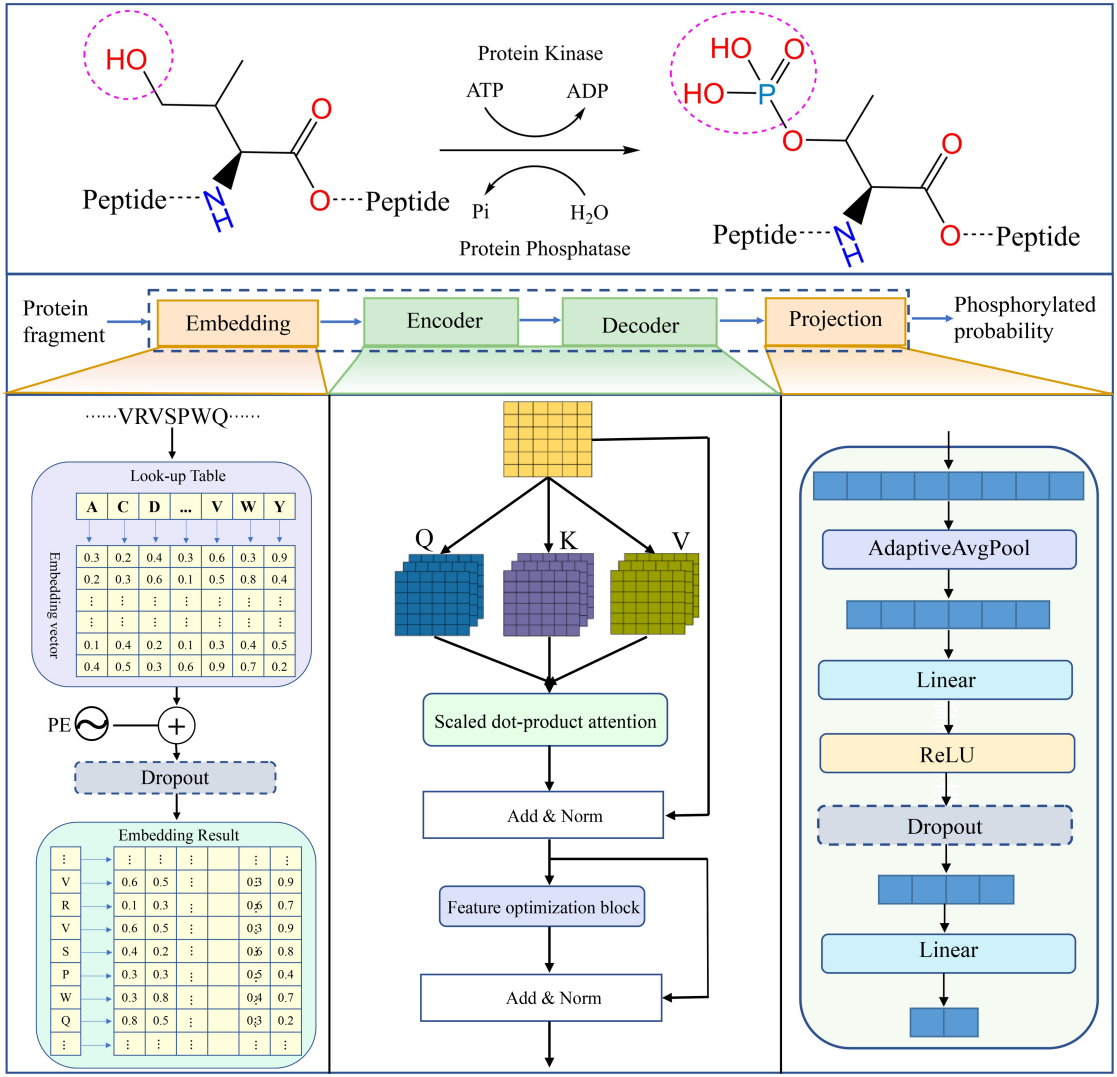
Framework of PSPred-ALE for SARS-CoV-2 phosphorylation modification site identification

Severe acute respiratory syndrome coronavirus 2 (SARS-CoV-2) is an enveloped positive-sense RNA virus that is closely related to SARS-CoV and several SARS-related coronaviruses (Lai *et al.* 2020, Zhou *et al.* 2020). The pathophysiological features of SARS-CoV-2 include acute respiratory distress and can lead to respiratory failure, multiorgan failure, and death (Herold *et al.* 2020). It has had a significant impact on human health and the global socioeconomic since its emergence in 2019 (Wolf *et al.* 2023). To develop antiviral therapies, scientists have recently used phosphoproteomic approaches to study the molecular mechanisms of SARS-CoV-2 infection by quantifying changes in protein abundance and phosphorylation (Bouhaddou *et al.* 2020, Hekman *et al.* 2020). Analysis of phosphorylation events after host infection may reveal the drug targets with therapeutic potential (Ochoa *et al.* 2016, 2020). Phosphorylation is a critical reversible post-translational modification (PTM) in proteins that regulates many essential processes in eukaryotes and prokaryotes. These processes include muscle contraction, neural activity, cell proliferation, cell signaling, differentiation, and development (Humphrey *et al.* 2015, Ardito *et al.* 2017, Takeuchi *et al.* 2017). Phosphorylation occurs when a phosphate group is covalently added to specific amino acid residues, such as serine (S), as shown in Fig. 1. This phosphorylation causes the protein to become charged, thereby altering the protein's structure, activities, and function (Huang *et al.* 2018). Therefore, the identification of this PTM in SARS-CoV-2 infection is crucial and can offer valuable insights into the infection mechanism, facilitating the development of essential drugs and therapeutic strategies (Gordon *et al.* 2020, Smith and Smith 2020).

Although wet experiments such as mass spectrometry can identify phosphorylation modification sites in high throughput, most laboratories do not have the necessary instruments and knowledge to use this technique (Steinke and Cook 1995, Beausoleil *et al.* 2006, Lyu *et al.* 2018). In addition, experimental techniques usually involve the utilization of expensive equipment and are always labor intensive. Consequently, using machine learning approaches to solve these problems has becoming popular due to its natural advantages. While a few computational models have been proposed for this purpose in recent years (Wang *et al.* 2017, Wang *et al.* 2020, Guo *et al.* 2021), only one machine learning predictor called DeepIPs, has been specifically designed to predict phosphorylation sites in host cells infected with SARS-CoV-2 (Lv *et al.* 2021). This predictor uses a CNN-LSTM based deep learning framework to make the prediction. The prediction accuracy of this model is about 80%. One possible reason is that current feature extraction methods are insufficient to extract more sequence information for machine learning algorithms to learned. Therefore, although this work greatly promoted the research on predicting SARS-CoV-2 phosphorylation modification sites, there is still an urgent need to explore and develop higher-performance predictors.

Here, we first demonstrate that SARS-CoV-2 infection does result in changes in phosphorylation modifications through bioinformatic analysis of A549 cells infected with SARS-CoV-2. Subsequently, we proposed PSPred-ALE, a novel SARS-CoV-2 phosphorylation modification site prediction model based on self-adaptive learning embedding. Our PSPred-ALE is a fully end-to-end architecture that does not require any feature engineering and can automatically learn and extract contextual information from sequences based on the embedding algorithm. Specially, the multihead attention mechanism is also applied to capture the global information in protein sequences and facilitates the model's understanding of discriminative features. On the other hand, we also investigated the performance of four conventional machine learning algorithms and 20 commonly used protein feature extraction methods. Comprehensive comparative experiments demonstrated that PSPred-ALE outperforms the state-of-the-art (SOTA) SARS-CoV-2 phosphorylation modification site predictors and handcrafted feature-based methods. This reveals that the features learned by the adaptive learning embedding mechanism are more effective and potentially than that of handcrafted feature engineering. Moreover, we also provide a 2D visualization comparison between different feature representation spaces to illustrate their efficacy in separating positive and negative samples. These experiments mentioned above also demonstrate the robustness and generalization of PSPred-ALE. We anticipate that this model will promote the exploration of new phosphorylation modification sites in SARS-CoV-2 infection and the understanding of the related pathogenesis and therapeutic strategies.

## 2 Materials and methods

### 2.1 Bioinformation analysis methods

RNA sequencing data (GSE 184536) for bioinformation analysis were downloaded from the GEO databases (https://www.ncbi.nlm.nih.gov/geo). The dataset contains independent biological triplicates of transformed lung alveolar (human A549) cells infected with SARS-CoV-2 (USA-WA1/2020, MOI: 2) for 2/6/12/24 h. Differentially expressed genes (DEGs) were analyzed by the R/limma package. To obtain significant DEGs, |LogFC > 0.1| and *P*-value of .01 were set as the thresholds. Pathway enrichment and analysis of biological processes (BPs) and Kyoto Encyclopedia of Genes and Genomes (KEGG) were performed by the R/clusterProfiler package based on the two databases of the KEGG and Gene Ontology (GO). *P* < .05 and enriched gene numbers (count) ≥2 were considered for the edges. Finally, the heatmap was used to show the results of the enrichment analysis. According to the results of GO analysis, experiments are differentiated into two groups (the high expression group and the low expression group). Then the gene set enrichment analysis (GSEA) (Subramanian *et al.* 2005) was performed by the R/clusterProfiler package and the R/biomaRt package in the viral infection group compared with the control groups, respectively. We computed the consistency *P*-value for each gene set, and *P*-value <.05 was considered to be enrichment significant. The enrichment curves of multiple functional groups were shown by R/GenePlot2 package, and their *P*-values were labeled. The association between infection time and BPs was analyzed using GSEA.

### 2.2 Datasets

The prediction of phosphorylation sites can be conceptualized as a binary classification task, where each specific residue is classified as a phosphorylated or nonphosphorylated site. When handling such classification tasks, a meticulously curated dataset that contains both positive and negative data is the foremost and critical factor to consider. In this work, we adopt the dataset collected by Lv et al. (2021) for training and evaluating machine learning models. Specifically, the construction of this dataset mainly includes the following steps to obtain a high-quality benchmark dataset: (i) a total of 14119 experimentally verified phosphorylation sites were collected from the literature, which were identified in human A549 cells infected with SARS-CoV-2 (Stukalov *et al.* 2021). These sites were carefully curated to ensure accuracy and reliability; (ii) the CD-HIT software (Li and Godzik 2006) was used to reduce the sequence redundancy and avoid model overfitting with the identity threshold value of 0.3; (iii) the processed sequences were truncated into peptide segments with serine/threonine (S/T) or tyrosine (Y) located at the center. If the central amino acid of a fragment is phosphorylated, the fragment is defined as a positive sample; otherwise, it is labeled as a negative sample. In this study, we only focused on predicting phosphorylation on the S/T due to insufficient samples of Y phosphorylation. A sequence having a phosphorylation site can be represented using following sequence structures: $S\left(p\right)=A_{-n}A_{-(n-1)}\ldots A_{-2}A_{-1}P\;A_{+1}A_{+2}\ldots A_{+(n-1)}A_{+n}$. Here, the highlighted letter P denotes the S/T at the positive phosphorylation site, while $A_{n}$ denotes the neighboring amino acids of the central phosphorylation site. The symbol “n” indicates the position of a given amino acid relative to the central residue, where $A_{-n}$ and $A_{+n}$ are the nth residues on the left and right sides of the positive site, respectively. Here, the segment sequence length ψ is uniformly 33 for both positive samples and negative samples; (iv) to solve the imbalance problem, the same number of negative samples as positive samples are randomly selected from all negative samples. The final dataset contains 10 774 samples, which can be expressed in a concise notation as follows: $D=D^{+}\cup D^{-}$, where $D^{+}$ and $D^{-}\;$represent positive and negative samples respectively, both of which contain 5387 sequences. (v) Eighty percent of the samples were used for training and validation of the models, and the remaining 20% were used as completely independent blind tests.

### 2.3 Model overview

The fundamental concept behind PSPred-ALE is the utilization of adaptive learning embeddings and self-attention mechanisms (Vaswani *et al.* 2017). This is achieved through the implementation of several techniques aimed at constructing and optimizing the model, which comprises four sub-modules, including the embedding layer, encoder layer, decoder layer and projection layer. The encoder layer and decoder layer share the same structure. The hyperparameters for the proposed PSPred-ALE model can be found in Supplementary Table S1. The main modules are described in detail as follows:

1. Embedding layer: The core idea of this module is to map each amino acid residue to a unique randomly initialized low-dimension and dense vector that can be learned and adjusts adaptively during model training via backpropagation. Thus, the whole peptide segment can be represented by a unique matrix through embedding layer. However, the above embedding methods do not consider the order of amino acids in the protein sequence, which is crucial for structure and function. Thus, the positional embedding was used to encode the amino acid position, which will provide additional information about the amino acid order of the protein sequence. For the amino acid at the p position in the sequence, the positional embedding is represented as a $d_{k}$-dimensional vector. The ith element PE(p)i of this vector can be expressed as follows:
  (1) $$\mathrm{PE}(p{)}_{2i}=\sin\left(p/10\;000^{2i/d_{k}}\right)$$
  (2) $$\mathrm{PE}(p{)}_{2i+1}=\cos\left(p/10\;000^{2i/d_{k}}\right),$$
  where 2i and 2i+1 represent the even and odd dimensions, respectively. The use of positional embedding allows us to capture both absolute and relative positional information of the amino acids in the sequence. To obtain the final embedding for the entire protein sequence, we add the amino acid embedding and the corresponding positional embedding together. This combined embedding represents the entire sequence and captures both the amino acid identities and their relative positions in the sequence. By incorporating both types of embeddings, the model can effectively capture the sequential information in the protein sequence and use it to make predictions.
2. Encoder layer: The encoder block takes as input the embedding vectors produced by the embedding layer. It comprises two key components: a multihead attention block and a feature optimization block. The encoder layer serves as the core of our model, enabling the embedding vectors to capture the context of each residue at different positions, and focus on relevant information while minimizing the impact of irrelevant information. The multihead attention mechanism comprises several self-attentions and the mathematical formulation for self-attention is as follows:
  (3) $$\left\{\begin{array}{c} Q=XW^{Q}\\ K=XW^{K}\\ V=XW^{V} \end{array}\;\right.$$
  (4) $$\mathrm{Self}-\mathrm{Attention}\left(Q,\;K,\;V\right)=\mathrm{softmax}\left(\frac{QK^{T}}{\sqrt{d_{k}}}\right)V,$$
  where $X\in R^{L\times d_{m}}$ is embedding layer output matrix, $d_{m}$ is the embedding dimension and L is the input sequence length. Q, *K*, $V\;\in R^{L\times d_{k}}$ are the query, key and value, respectively, which are obtained by applying linear transformations of X with weight matrices $W^{Q}$, $W^{K}$, $W^{V}\;\in R^{d_{m}\times d_{k}}$. $d_{k}$ is the dimension of the query, key and value vector. Both $d_{m}$ and $d_{k}$ are hyperparameters that need to be set manually.The feature optimization block consists of fully connected layers, with the number of channels gradually increasing and then decreasing. This block is designed to ensure that the input and output dimensions are consistent, and to obtain a better feature representation.
3. Projection layer: The last module of our model is referred to as the projection layer, which is composed of fully connected layers and nonlinear activation functions. The feature matrix obtained from the previous layer represents the input sequence, with each column corresponding to the context vector of a specific residue. To obtain the learned representations of the entire sequence, we reshape the feature matrix into a 1D feature vector. This flattened feature vector is then passed through the projection layer, which transforms the sequence representations into a probabilistic distribution of classes. By computing the probabilities for each class, we can determine the likelihood of the central amino acid of a fragment being a phosphorylation site or a nonphosphorylation site.

### 2.4 Implementation of traditional machine learning models

To use machine learning algorithms implementation for distinguishing protein phosphorylation sites, it is necessary to convert biological sequence data into numeric feature vectors through various encoding methods, since these algorithms cannot process the amino acid sequences directly. This process of transforming the sequences into mathematical expressions that accurately capture the intrinsic correlations with the desired targets is called feature extraction or feature encoding (Liu 2019). The typical studies of phosphorylation site prediction heavily use traditional machine learning algorithms and handcrafted feature extraction methods to build prediction tools. However, no studies have specifically examined the effectiveness of classical protein features and conventional machine classifiers in identifying phosphorylation sites associated with SARS-CoV-2 infection thus far. Therefore, to gain a better understanding of the most effective methods for representing phosphorylation site protein sequences, we have undertaken a comprehensive study that compares an adaptive learning embedding features-based model with handcrafted features-based models. By exploring both approaches, we can better determine which method is more effective in predicting phosphorylation sites in SARS-CoV-2 infection, and thus provide insights into how to optimize computational tools for studying protein modification sites. Comparing the performance of different methods can be a challenging task when there are a large number of handcrafted features to consider. To address this issue, we have chosen to focus on twenty of the most popular statistical features for prediction and analysis. These features include amino acid composition (AAC), AAindex, learn from alignments (AESNN), amphiphilic pseudo-amino acid composition (APAAC), adaptive skip dipeptide composition (ASDC), BLOSUM62 (BLOSUM), composition of k-Spaced amino acid group pairs (CKSAAGP), composition (CTDC), k-spaced conjoint triad (CTriad), dipeptide deviation from expected mean (DDE), PseAAC of distance-pairs and reduced alphabet (DP), di-peptide composition (DPC), enhanced amino acid composition (EAAC), enhanced grouped amino acid composition (EGAAC), k-spaced conjoint triad (KSCT), overlapping property features (OPF), pseudo-amino acid composition (PAAC), quasi-sequence-order (QSOrder), ZScale, and composition of k-spaced amino acid pairs (CKSAAP). We have selected four of the most commonly used classification algorithms in biological sequence analysis and prediction to build models. These algorithms include Support Vector Machine (SVM), Random Forest (RF), Light Gradient Boosting Machine (LGBM), and eXtreme Gradient Boosting (XGBT). These algorithms were chosen based on their well-established performance in the field and their ability to effectively handle complex and high-dimensional data, such as protein sequences. To implement and compare the different features and classifiers mentioned above, we have utilized the iLearn package (Chen *et al.* 2021) for extracting the statistical features and the scikit-learn API (Swami and Jain 2013) for implementing the traditional machine learning algorithms. Grid search was used to fine-tune the hyperparameters of the classifiers, and the search range is provided in Supplementary Table S2.

### 2.5 Performance evaluation strategies

We selected five commonly used metrics for evaluating the performance of binary classification models, namely, accuracy (ACC), sensitivity (SE), specificity (SP), area under the receiver operating characteristic curve (AUC), and Matthew's correlation coefficient (MCC). Detailed descriptions and calculation formulas for these metrics are presented in the supplementary material (Evaluation metrics section).

## 3 Results and discussion

### 3.1 Bioinformation analysis of A549 cells with SARS-CoV-2 infection

**Figure 2.**
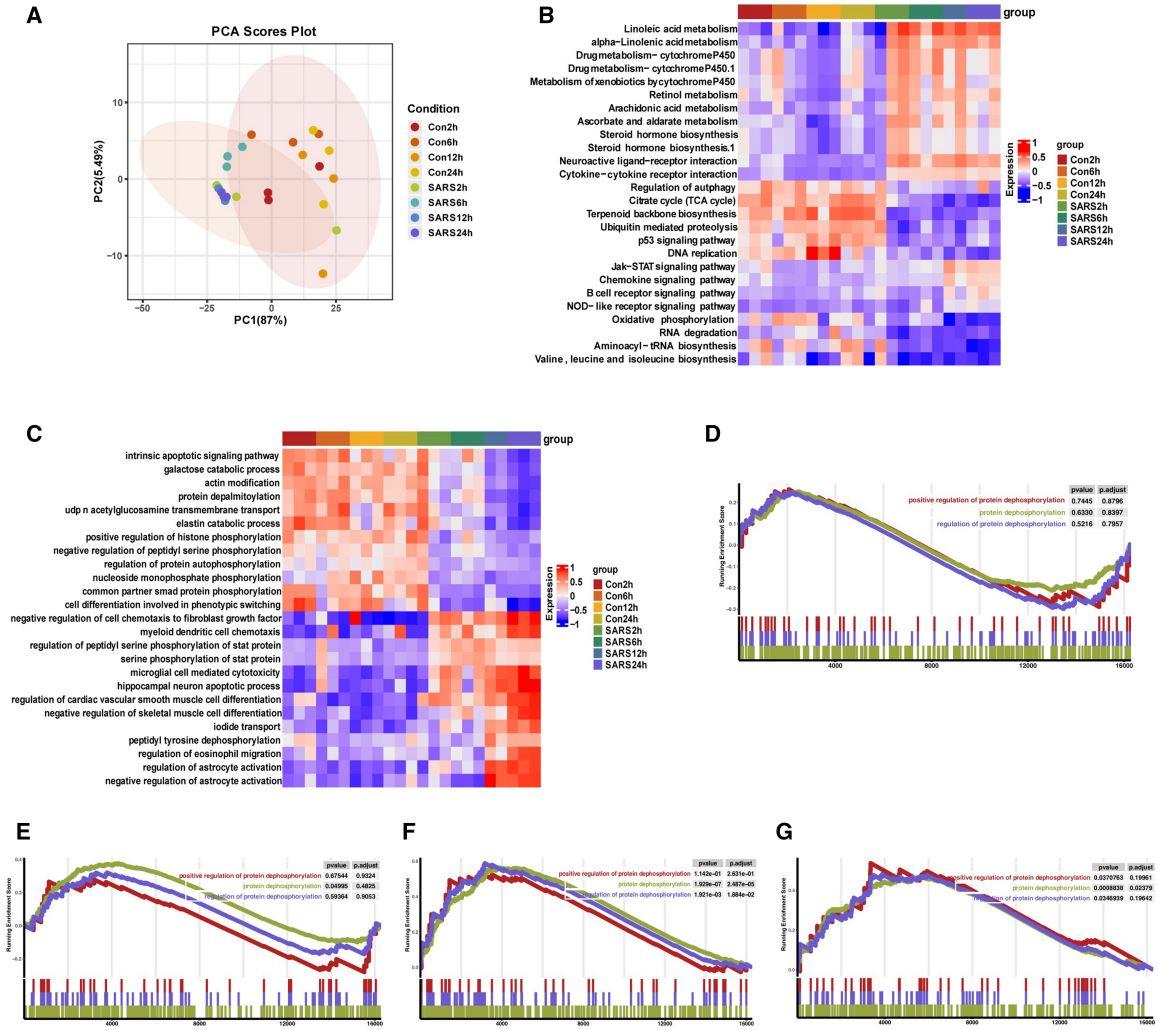
(A) PCA of A549 cells infected with SARS-CoV-2 at different hours post-infection (hpi) compared to uninfected cells. (B–C) Heatmap of the top 26 KEGG pathways (B) and top 25 GO pathways (C) in SARS-CoV-2 infection groups and uninfected groups at different time course of post-infection. GSEA was performed against the KEGG datasets or GO dataset for BPs. The color of the lattices represents the false discovery rate value for each enriched KEGG term or GO term. (D–G) Enrichment analysis of GO involved the dephosphorylation pathway. GSEA of 2 hpi (D), 6 hpi (E), 12 hpi (F), 24 hpi (G), *P*-value and *P*-adjusted are indicated. Con2h, mock-infected A549 cells (2 hpi); SARS2h, SARS-CoV-2-infected A549 cells (2 hpi); Con6h, mock-infected A549 cells (6 hpi); SARS6h, SARS-CoV-2-infected A549 cells (6 hpi); Con12h, mock-infected A549 cells (12 hpi); SARS12h, SARS-CoV-2-infected A549 cells (12 hpi); Con24h, mock-infected A549 cells (24 hpi); SARS24h, SARS-CoV-2-infected A549 cells (24 hpi)

We performed principal component analysis (PCA) on the mRNA dataset to reduce the dimensionality of the data and visualize sample differences between infected and uninfected groups. The PCA showed that the eigenvalues of the two first principal components represented 92.49% of the total variance (PC1: 87%; PC2: 5.49%) of the observations (Fig. 2A). Figure 2A also shows significant segregation of the infected and uninfected groups along the second dimension, which suggests that human lung epithelial A549 cell infection with the novel coronavirus will have an impact on host cells, especially 24 h after infection. We concentrate on the BPs occurring across various time scales following infection in order to obtain understanding of potential cellular function alterations. To find the genes implicated in significantly altered pathways, we first mapped all the DEGs in the KEGG database. At 12 and 24 h after infection, it revealed a high enrichment of immune-related pathways (Fig. 2B). Moreover, GO enrichment analysis revealed that most phosphorylation-related pathways were more enriched in uninfected groups than in infected groups (Fig. 2C). During the stage of SARS-CoV-2 infection, phosphorylation signaling stands in place of transcriptional control as the main host defense mechanism (Bouhaddou *et al.* 2020). The GSEA results showed no significant changes in protein phosphorylation pathway in A549 cells after 2 and 6 h of virus infection (Fig. 2D and E), but significant changes in protein phosphorylation pathway after 12 and 24 h of infection, which is highly suggestive of self-defense of host lung epithelial cells starting 12 h after virus infection (Fig. 2F and G). Here, we investigate the relationship between viral infection and host response, demonstrating significant variations in immune-related pathways, metabolic pathways, and protein phosphorylation events in host cells. Consistent with reports in the literature (Nilsson-Payant *et al.* 2021), the transcriptional response to the virus peaked at 12 h post infection and then increased steadily until 24 h post infection.

### 3.2 Performance comparison of PSPred-ALE with existing predictors

**Figure 3.**
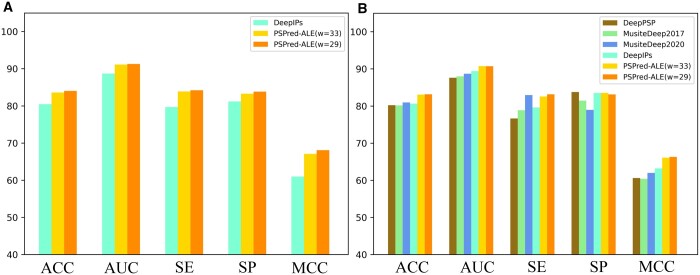
(A) and (B) Performance comparison between PSPred-ALE and existing predictors using 5-fold cross validation and an independent test, respectively

To evaluate the proposed PSPred-ALE, we compared its performance with four SOTA prediction tools, including DeepPSP, MusiteDeep2017, MusiteDeep2020, and DeepIPs. The performance metrics of MusiteDeep2017, MusiteDeep2020 and DeepPSP were obtained by Lv *et al.* by rebuilding the models. Thus, all compared models are compared fairly based on the same dataset with a sequence length of 33. The performance results of these compared predictors are summarized in Table 1 and shown in Fig. 3.

**Table 1.** Comparison of the proposed PSPred-ALE and existing methods on training and testing datasets.

Model	5-Fold cross validation					Independent testing				
	ACC (%)	AUC	SE (%)	SP (%)	MCC	ACC (%)	AUC	SE (%)	SE (%)	MCC
DeepPSP						80.21	0.876	76.65	83.78	0.606
MusiteDeep2017						80.17	0.880	78.87	81.46	0.604
MusiteDeep2020						80.95	0.887	82.95	78.96	0.620
DeepIPs	80.45	0.887	79.70	81.19	0.610	80.63	0.894	79.61	83.50	0.632
Ours (ψ = 33)	83.58	0.911	83.89	83.26	0.671	83.06	0.907	82.58	83.54	0.661
Ours (ψ = 29)	84.03	0.913	84.22	83.84	0.681	83.14	0.907	83.15	83.13	0.663

As evidenced by Fig. 3A, our newly proposed PSPred-ALE exhibits significantly superior performance than the current SOTA predictor DeepIPs, when evaluated using 5-fold cross validation. Specifically, the ACC, AUC, SE, SP, and MCC values are about 3.13%, 2.40%, 4.19%, 2.07%, and 6.12% higher than those of DeepIPs, respectively. To better evaluate model's performance, it's important to compare it with other existing phosphorylation site prediction tools using independent test data. This allows for a more comprehensive assessment of its accuracy and robustness. As shown in Table 1 and Fig. 3B, our model achieved overall better performance on the independent dataset. Specifically, the ACC, AUC, and MCC of our model is about 2.1%–2.9%, 1.3%–3.1%, and 2.9%–5.7% higher than those of other three predictors, respectively. Although our model's SE and SP values are not the highest, they are only slightly worse than the best. It is worth noting that the SE and SP values of our model are similar, both at about 83%, while the gap between these two indicators of existing predictors is very large, with the largest being close to 7.1%. This means that our model is more balanced in its ability to identify both negative and positive samples. To this end, the results demonstrate that our PSPred-ALE is superior to the SOTA approaches for the identification of phosphorylation sites related to the SARS-CoV-2 infection.

### 3.3 Window size optimization

**Figure 4.**
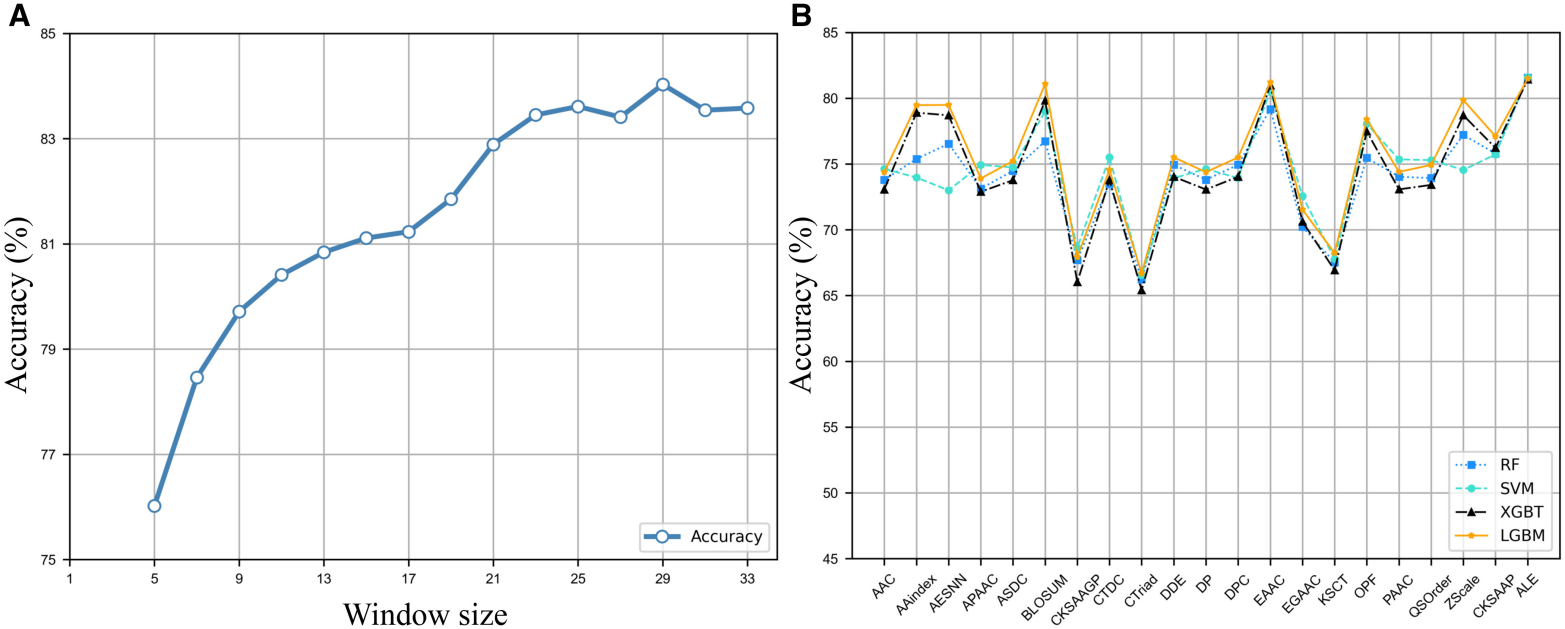
(A) The impact of the window size on the model’s performance. (B) The performance comparison of twenty handcrafted features and adaptive learning embedding features on four traditional machine learning classifiers

To accurately identify phosphorylation sites, it is important to consider the bias of amino acids specific to their positions relative to the phosphorylation site. This requires determining the optimal window for flanking sequences around phosphorylation sites. On the other hand, the performance of the predictive model is strongly related to the input sequence length. We analyzed the impact of window size ψ on the predictive performance using the training dataset. The scope of w ranged from 5 to 33, with an increment of 2 amino acids. Figure 4A shows the predictive accuracies obtained through a 5-fold cross validation test for models using different window sizes. When the window size is between 5 and 23, the model's performance increases dramatically with the window size. When it reaches 29, the model's performance no longer improves with the window change. ψ = 29 was chosen as it corresponded to the maximum accuracy value achieved through 5-fold cross validation. So, we use a sequence length of 29 for the next step analysis. The shorter sequence of inputs means a lower consumption of computing resources. To further demonstrate the efficacy of PSPred-ALE, independent testing was conducted, yielding an ACC of 83.14%, AUC of 0.907, SE of 83.15%, SP of 83.13%, and MCC of 0.663. All these results are also summarized in Table 1 and presented in Fig. 3B.

### 3.4 Conventional machine learning models using handcrafted features

All compared models were performed and evaluated on the training dataset, with a 5-fold cross validation approach. Figure 4B presents the predictive performances of the compared models, and Supplementary Table S3 provides more detailed results. The following observations can be made based on the results.

As we can see from Fig. 4B, the performance of handcrafted feature extraction methods varies greatly. While some encodings may produce highly accurate predictors, others may perform poorly and yield less reliable results. Overall, the four classifiers share the same pattern on different features, where the LGBM classifier tended to perform the best, while the RF classifier tended to perform the worst. Regarding the different feature encoding methods, some of them consistently perform well across all four algorithms. For example, EAAC performs well across all four machine learning algorithms but particularly well with LGBM and XGBT. The BLOSUM achieves the second-highest accuracy on four classifiers except RF. On the other hand, some feature extraction methods consistently perform poorly across all machine learning algorithms, such as CTriad, which has the lowest cross validation accuracy for all four algorithms. Specially, the EAAC and BLOSUM on the LGBM classifier achieved scores of 81.49% and 81.08%, respectively, which is about 1.04% and 0.63% higher than that of DeepIPs, respectively. This also proves that the feature representation method used by DeepIPs has no advantage over handcrafted features. In summary, the results suggest that the choice of feature extraction method and machine learning algorithm can have a significant impact on the performance of a protein classification task, and that a combination of EAAC or BLOSUM and LGBM may be a particularly effective approach for this dataset.

### 3.5 Conventional machine learning models using adaptive learning embedding features

To gain a more intuitive understanding of the effectiveness of the adaptive learning embedding features, we further utilized them to train traditional machine learning models. We output the learned representation matrix before the fully connected layer and flatten for each sample. Then, these features were input to the classifiers to obtain corresponding models. The results metrics are also presented in Supplementary Table S4 and plotted in Fig. 4B. We can see that the adaptive learning embedding features (ALE) learned by our method outperform all the statistical-based traditional handcrafted features on the traditional classifiers. Notably, the results of the adaptive learning embedding features on the four classifiers are very close, all at about 81.5%, which is better than DeepIPs and all handcrafted features. Comprehensively, the adaptive learning embedding features is better and more stable for representing the protein sequence in this study.

### 3.6 Feature visualization comparison by dimension reduction

**Figure 5.**
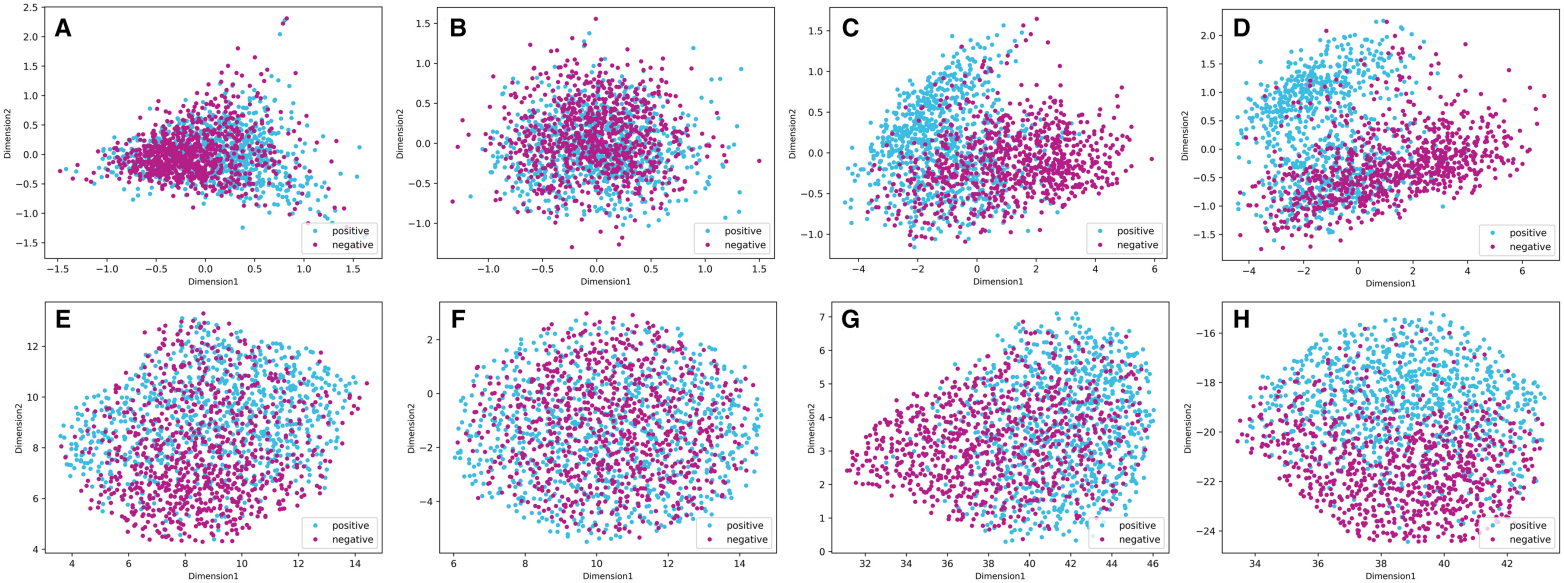
Dimension reduction of samples by PCA and UMAP. (A) and (E) are the PCA and UMAP visualizations of the handcrafted feature EAAC, respectively. (B–D) are the PCA visualizations of adaptive learning embedding features during the training process at 1, 20, and 100 epochs, respectively. (F–H) are the UMAP visualizations of adaptive learning embedding features during the training process at 1, 20, and 100 epochs, respectively

To provide an intuitive demonstration of the effectiveness of PSPred-ALE, we reduced the feature space to a 2D space using principal component analysis (PCA) (Shlens 2014) and uniform manifold approximation and projection (UMAP) (Mcinnes *et al.* 2018) on training datasets. This allows us to gain insights into the learned features and how they contribute to the model's predictions. The resulting plots are shown in Fig. 5. PCA is a well-established and widely used linear method for dimension reduction, while UMAP is a newer, more advanced nonlinear method that is particularly effective at preserving the structure and relationships between data points in high-dimensional space. As we can see from Fig. 5, the PCA and UMAP plots show similar patterns. For handcrafted feature EAAC, although it can also be seen that some samples are aggregated, most of them are still mixed (Fig. 5A and E). For PSPred-ALE, the positive and negative samples are mixed before training because the embeddings are initialized randomly. This demonstrated that the model lacked distinguishing ability at this stage. As the number of training epochs increases, the negative and positive points are gradually distinguished from each other (Fig. 5C and G). After training, there is an obvious gap between positive sample clusters and negative sample clusters in the PCA space (Fig. 5D). The UMAP space also shows a clear trend where the positive samples tend to be distributed on the upper side, while the negative samples tend to be distributed on the lower side, and there is also an obvious boundary in the middle (Fig. 5H). Both methods indicate that our model indeed learns some distinguishable features better than the handcrafted features after training to separate two classes of samples. Meanwhile, a considerable portion of the samples fall in the opposing regions, which explains why our model still has a 17% error rate to some degree. We conjecture that the positive samples that were incorrectly classified as negative by our method may possess certain features that were not captured by our model. Thus, it is necessary to conduct further investigation into the unique sequence patterns and properties of these indistinguishable samples in the future. Overall, the results of the dimensionality reduction analysis provide additional evidence that our model is effective at accurately classifying samples and capturing important information from the input raw protein sequences.

## 4 Conclusion

The identification of SARS-CoV-2 phosphorylation modification sites is a significant endeavor that can facilitate the development of related new drugs and treatment strategies, which indirectly contribute to global health care. According to the results of the bioinformatics analysis, it is clear that protein phosphorylation events are significantly altered upon cellular infection with SARS-CoV-2. However, the current lack of machine learning tools specifically designed for identifying these modification sites poses a challenge in this field. In this work, we developed a novel deep learning predictor named PSPred-ALE for identifying SARS-CoV-2 phosphorylation modification sites. The framework utilizes only the protein primary sequence for prediction. In particular, we use an adaptive learning embedding algorithm to generate better protein sequence representations, which can overcome the inefficiencies of traditional computational methods that rely on handcrafted feature engineering. The experimental results demonstrate that our model is capable of adaptively extracting high-quality and discriminative features from different class examples, resulting in a significant improvement in prediction performance. The comparative experiments show that PSPred-ALE achieves superior performance on most evaluation metrics when compared to existing methods, thereby providing further evidence that protein sequences themselves contain sufficient information to predict SARS-CoV-2 phosphorylation modification sites. To facilitate use by the relevant research community, we have made the source code for implementing PSPred-ALE publicly available. Due to the current lack of accurate models for predicting phosphorylation modification sites of SARS-CoV-2, our study presents a comprehensive methodology that can serve as a foundation for future research in this field. We expect that PSPred-ALE will be a valuable tool to complement wet lab experiments in identifying phosphorylation modification sites of SARS-CoV-2 infection, and its application can help reveal relevant biological functional mechanisms and perform numerous sequence-based analyses.

## Supplementary Material

btad627_Supplementary_Data
