# Supplementary material for: Adaptive learning embedding features to improve the predictive performance of SARS-CoV-2 phosphorylation sites
Source: Bioinformatics. 2023 Oct 17;39(11):btad627. doi: 10.1093/bioinformatics/btad627 (PMC10628388; doi:10.1093/bioinformatics/btad627)
Supplement: btad627_Supplementary_Data [file btad627_supplementary_data.docx]

**Adaptive learning embedding features to improve the predictive performance of SARS-CoV-2 phosphorylation sites**

Shihu Jiao^1^, Xiucai Ye^1,*^, Chunyan Ao^2^, Tetsuya Sakurai^1^, Quan Zou^2,3^, Lei Xu^4,*^

1. Department of Computer Science, University of Tsukuba, Tsukuba 3058577, Japan
2. Institute of Fundamental and Frontier Sciences, University of Electronic Science and Technology of China, Chengdu, China
3. Yangtze Delta Region Institute (Quzhou), University of Electronic Science and Technology of China, Quzhou, China
4. School of Electronic and Communication Engineering, Shenzhen Polytechnic, Shenzhen, China

*Corresponding author: Xiucai Ye: yexiucai@cs.tsukuba.ac.jp; Lei Xu: csleixu@szpt.edu.cn.

**Contents**

**1 Evaluation metrics** P2

**2 Supporting Tables**

Table S1 Hyperparameters values of PSPred-ALE P3

Table S2 Hyperparameters search range for four traditional classifiers P3

Table S3 Five-fold cross validation results of the twenty handcrafted features on four traditional machine learning classifiers P3-P5

Table S4. Five-fold cross validation results of adaptive learning embedding features on traditional classifiers. P5

1. **Evaluation metrics:**

There are various metrics or evaluation measures used to assess the quality of a model. Here are five commonly used evaluation metrics: ACC (accuracy), SE (sensitivity), SP (specificity), MCC (Matthew's correlation coefficient), and area under the receiver operating characteristic (ROC) curve (AUC). The formulas and detailed descriptions of these metrics are summarized in the supporting material.

$$\left\{ \begin{aligned} A\mathrm{CC}= \frac{TP+TN}{TP+FP+TN+FN}*100\% (1) \\ SE= \frac{TP}{TP+FN}*100\% (2) \\ SP= \frac{TN}{TN+FP}*100\% (3) \\ \begin{matrix} MCC=\frac{(TP\times TN)-(FP\times FN)}{\sqrt{(TP+FP)\times(TN+FN)\times(TP+FN)\times(TN+FP)}} (4) \end{matrix} \end{aligned} \right.$$

where "true positive" (TP) refers to the count of positive instances that have been accurately identified, "true negative" (TN) refers to the count of negative instances that have been accurately identified, "false positive" (FP) refers to the count of negative instances that have been inaccurately identified as positive, and "false negative" (FN) refers to the count of positive instances that have been inaccurately identified as negative. ACC measures the proportion of correct predictions made by the model over all predictions. SE measures the proportion of actual positive instances that are correctly predicted as positive by the model. SP measures the proportion of actual negative instances that are correctly predicted as negative by the model. MCC is a correlation coefficient between the predicted and true binary classifications, considering true and false positives and negatives. The AUC metric is calculated as the area enclosed by the Receiver Operating Characteristic (ROC) curve and the x-axis, where the false-positive rate (FPR) is represented on the x-axis and the true positive rate (TPR) is represented on the y-axis. AUC measures the model's ability to distinguish between positive and negative instances, by calculating the area under the ROC curve.

1. **Supporting Tables**

Table S1. Hyperparameters values of PSPred-ALE.

| **Layers** | **Parameter** |
| --- | --- |
| Batch size | 256 |
| Learning rate | 0.0005 |
| Dimension of adaptive embedding | 16 |
| Feature optimization block | 32 |
| Dimension of K and V | 16 |
| Number of heads of multi-head self-attention | 8 |
| Number of layers of multi-head self-attention | 1 |
| 1D adaptive average pooling | 128 |
| Linear1 in projection layer | 64 |
| Linear2 in projection layer | 2 |
| Dropout in projection layer | 0.5 |

Table S2. Hyperparameters search range for four traditional classifiers.

| **Method** | **Parameter*** | **Tested values** |
| --- | --- | --- |
| RF | n_estimators | 10–200 with an interval of 20 |
| SVM | C | [2^-5^–2^15^] in log_2_ steps |
|  | gamma | [2^-15^–2^-5^] in log_2_ steps |
| XGBT | n_estimator | 10-200 with an interval of 20 |
|  | learning_rate | 0.001-0.5 with an interval of 0.1 |
| LGBM | n_estimator | 10-200 with an interval of 20 |

^*^Parameter name in the Scikit-learn implementation.

RF: Random Forest, SVM: Support Vector Machine, XGBT: eXtreme Gradient Boosting, LGBM: Light Gradient Boosting Machine.

Table S3: Five-fold cross validation results of the twenty handcrafted features on four tradition machine learning classifiers.

| **Feature name** | **Classifier** | **ACC (%)** | **AUC** | **SE (%)** | **SP (%)** | **MCC** |
| --- | --- | --- | --- | --- | --- | --- |
| **AAC** | RF | 73.80 | 0.808 | 77.34 | 70.26 | 0.477 |
|  | SVM | 74.63 | 0.815 | 79.97 | 69.29 | 0.495 |
|  | XGBT | 73.06 | 0.801 | 76.51 | 69.61 | 0.462 |
|  | LGBM | 74.38 | 0.813 | 78.55 | 70.22 | 0.489 |
| **AAindex** | RF | 75.38 | 0.838 | 68.15 | 82.61 | 0.513 |
|  | SVM | 73.97 | 0.816 | 74.63 | 73.31 | 0.479 |
|  | XGBT | 78.90 | 0.867 | 78.04 | 79.76 | 0.578 |
|  | LGBM | 79.47 | 0.872 | 78.32 | 80.62 | 0.590 |
| **AESNN** | RF | 76.54 | 0.840 | 69.41 | 83.68 | 0.536 |
|  | SVM | 73.00 | 0.802 | 74.42 | 71.59 | 0.460 |
|  | XGBT | 78.70 | 0.862 | 77.02 | 80.39 | 0.574 |
|  | LGBM | 79.49 | 0.871 | 77.92 | 81.06 | 0.590 |
| **APAAC** | RF | 73.13 | 0.802 | 74.95 | 71.31 | 0.463 |
|  | SVM | 74.93 | 0.817 | 79.99 | 69.87 | 0.501 |
|  | XGBT | 72.89 | 0.795 | 75.63 | 70.15 | 0.458 |
|  | LGBM | 73.90 | 0.808 | 77.62 | 70.17 | 0.479 |
| **ASDC** | RF | 74.48 | 0.814 | 78.09 | 70.87 | 0.491 |
|  | SVM | 74.74 | 0.816 | 79.85 | 69.64 | 0.497 |
|  | XGBT | 73.77 | 0.809 | 76.30 | 71.24 | 0.476 |
|  | LGBM | 75.22 | 0.822 | 79.02 | 71.43 | 0.506 |
| **BLOSUM** | RF | 76.73 | 0.848 | 69.29 | 84.17 | 0.541 |
|  | SVM | 79.00 | 0.868 | 78.34 | 79.67 | 0.580 |
|  | XGBT | 79.81 | 0.878 | 79.04 | 80.57 | 0.596 |
|  | LGBM | 81.08 | 0.888 | 79.92 | 82.24 | 0.622 |
| **CKSAAGP** | RF | 67.70 | 0.735 | 70.61 | 64.79 | 0.355 |
|  | SVM | 68.62 | 0.746 | 73.35 | 63.88 | 0.374 |
|  | XGBT | 66.02 | 0.714 | 68.69 | 63.35 | 0.321 |
|  | LGBM | 67.93 | 0.739 | 73.31 | 62.56 | 0.361 |
| **CTDC** | RF | 73.49 | 0.803 | 77.58 | 69.41 | 0.471 |
|  | SVM | 75.50 | 0.819 | 83.05 | 67.94 | 0.516 |
|  | XGBT | 73.77 | 0.801 | 78.46 | 69.08 | 0.477 |
|  | LGBM | 74.55 | 0.811 | 80.22 | 68.87 | 0.494 |
| **CTriad** | RF | 66.24 | 0.721 | 66.60 | 65.88 | 0.325 |
|  | SVM | 66.55 | 0.724 | 65.55 | 67.55 | 0.331 |
|  | XGBT | 65.41 | 0.709 | 66.69 | 64.14 | 0.308 |
|  | LGBM | 66.69 | 0.726 | 70.03 | 63.35 | 0.335 |
| **DDE** | RF | 74.94 | 0.820 | 77.18 | 72.70 | 0.499 |
|  | SVM | 73.93 | 0.817 | 74.68 | 73.19 | 0.479 |
|  | XGBT | 74.03 | 0.815 | 75.60 | 72.45 | 0.481 |
|  | LGBM | 75.50 | 0.826 | 77.97 | 73.03 | 0.511 |
| **DP** | RF | 73.80 | 0.808 | 77.34 | 70.26 | 0.477 |
|  | SVM | 74.63 | 0.815 | 79.97 | 69.29 | 0.495 |
|  | XGBT | 73.06 | 0.801 | 76.51 | 69.61 | 0.462 |
|  | LGBM | 74.38 | 0.813 | 78.55 | 70.22 | 0.489 |
| **DPC** | RF | 74.94 | 0.820 | 77.18 | 72.70 | 0.499 |
|  | SVM | 73.93 | 0.817 | 74.68 | 73.19 | 0.479 |
|  | XGBT | 74.03 | 0.815 | 75.60 | 72.45 | 0.481 |
|  | LGBM | 75.50 | 0.826 | 77.97 | 73.03 | 0.511 |
| **EAAC** | RF | 79.16 | 0.870 | 81.27 | 77.04 | 0.584 |
|  | SVM | 80.56 | 0.881 | 81.27 | 79.85 | 0.611 |
|  | XGBT | 80.97 | 0.889 | 80.78 | 81.15 | 0.619 |
|  | LGBM | 81.49 | 0.896 | 81.71 | 81.27 | 0.630 |
| **EGAAC** | RF | 70.22 | 0.770 | 70.96 | 69.48 | 0.404 |
|  | SVM | 72.56 | 0.796 | 76.32 | 68.80 | 0.453 |
|  | XGBT | 70.62 | 0.773 | 72.17 | 69.08 | 0.413 |
|  | LGBM | 71.53 | 0.783 | 74.03 | 69.03 | 0.431 |
| **KSCT** | RF | 67.51 | 0.736 | 68.25 | 66.78 | 0.350 |
|  | SVM | 67.78 | 0.745 | 66.06 | 69.50 | 0.356 |
|  | XGBT | 66.92 | 0.726 | 67.41 | 66.43 | 0.338 |
|  | LGBM | 68.23 | 0.750 | 69.99 | 66.48 | 0.365 |
| **OPF** | RF | 75.46 | 0.831 | 67.97 | 82.96 | 0.515 |
|  | SVM | 78.05 | 0.855 | 77.27 | 78.83 | 0.561 |
|  | XGBT | 77.47 | 0.850 | 76.58 | 78.37 | 0.550 |
|  | LGBM | 78.40 | 0.856 | 76.39 | 80.41 | 0.568 |
| **PAAC** | RF | 74.03 | 0.809 | 76.39 | 71.66 | 0.481 |
|  | SVM | 75.35 | 0.820 | 80.55 | 70.15 | 0.510 |
|  | XGBT | 73.07 | 0.800 | 75.51 | 70.64 | 0.462 |
|  | LGBM | 74.40 | 0.814 | 78.20 | 70.59 | 0.489 |
| **QSOrder** | RF | 73.93 | 0.809 | 77.65 | 70.22 | 0.480 |
|  | SVM | 75.29 | 0.820 | 79.99 | 70.59 | 0.508 |
|  | XGBT | 73.41 | 0.803 | 76.14 | 70.68 | 0.469 |
|  | LGBM | 74.93 | 0.815 | 78.71 | 71.15 | 0.500 |
| **Zscale** | RF | 77.21 | 0.851 | 70.29 | 84.12 | 0.549 |
|  | SVM | 74.55 | 0.817 | 74.35 | 74.74 | 0.491 |
|  | XGBT | 78.70 | 0.869 | 77.97 | 79.43 | 0.574 |
|  | LGBM | 79.86 | 0.878 | 78.74 | 80.99 | 0.597 |
| **CKSAAP** | RF | 75.79 | 0.828 | 79.92 | 71.66 | 0.518 |
|  | SVM | 75.73 | 0.826 | 77.34 | 74.12 | 0.515 |
|  | XGBT | 76.22 | 0.834 | 77.51 | 74.93 | 0.525 |
|  | LGBM | 77.10 | 0.842 | 79.60 | 74.61 | 0.543 |

Table S4. Performance results of adaptive learning embedding features on traditional classifiers.

| Classifier | ACC (%) | AUC | SE (%) | SP (%) | MCC |
| --- | --- | --- | --- | --- | --- |
| RF | 81.57 | 0.892 | 82.47 | 80.66 | 0.632 |
| SVM | 81.59 | 0.892 | 82.24 | 80.94 | 0.632 |
| XGBT | 81.42 | 0.892 | 82.17 | 80.67 | 0.632 |
| LGBM | 81.39 | 0.892 | 82.10 | 80.67 | 0.632 |
